# A Novel Approach for Foreign Substances Detection in Injection Using Clustering and Frame Difference

**DOI:** 10.3390/s111009121

**Published:** 2011-09-27

**Authors:** Guiliang Lu, Yu Zhou, Yao Yu, Sidan Du

**Affiliations:** School of Electronic Science and Engineering, Nanjing University, Nanjing 210093, Jiangsu, China; E-Mail: luguiliang@gmail.com (G.L.); coff128@nju.edu.cn (S.D.)

**Keywords:** computer vision, detection of foreign substances, clustering, frame difference

## Abstract

This paper focuses on developing a novel technique based on machine vision for detection of foreign substances in injections. Mechanical control yields spin/stop movement of injections which helps to cause relative movement between foreign substances in liquid and an ampoule bottle. Foreign substances are classified into two categories: subsiding-slowly object and subsiding-fast object. A sequence of frames are captured by a camera and used to recognize foreign substances. After image preprocessing like noise reduction and motion detection, two different methods, Moving-object Clustering (MC) and Frame Difference, are proposed to detect the two categories respectively. MC is operated to cluster subsiding-slowly foreign substances, based on the invariant features of those objects. Frame Difference is defined to calculate the difference between two frames due to the change of subsiding-fast objects. 200 ampoule samples filled with injection are tested and the experimental result indicates that the approach can detect the visible foreign substances effectively.

## 1. Introduction

Injection liquids and transfusion solutions have been widely used in clinical treatment in the world. Foreign substances, such as metal shavings, glass shavings and fibres, may appear in injections during the process of production but they are prohibited to appear in qualified injections. These foreign substances can cause serious diseases such as tumor, phlebitis, anaphylactic reaction or even death when they are injected into blood. Despite of such large production and strict standard of injection liquid, most of the pharmaceutical corporations in China still detect the foreign substances artificially [1]: an injection is illumined by a light source, a worker turns it over to checks whether visible foreign substances appear and decides the injection is qualified or not. Though this method is simple, it can result in low accuracy and low efficiency. It is significant to invent an automatic detection method with high accuracy.

Previous works based on machine vision are developed to detect foreign substances. A foreign substances inspection system can be traced back to 1970s [2]. A lot of work is done later. Researchers have developed detection algorithm based on a sequence of frames about the liquid. Ishii *et al.* [3] breaks up frames into odd ones and even ones, obtains the maximum and minimum values of pixel in odd and even frames respectively, and computes the difference between these values to detect foreign substances. Ishii’s method has a small amount of computation but may be confused by bubbles. Lu *et al.* [4] proposes another method which use Mean Shift to track the moving object and judge foreign substances by trajectory. Lu’s method can distinguish foreign substances and bubbles effectively but is weak in tracking fast-moving objects. Zhou *et al.* [5] depicts a classifier (SVM) to judge foreign substances and bubbles based on area size and ratio of length to width. This classifier has a better precision than Lu’s method but it mainly depends on samples. Moghadas *et al.* [6] uses MLP neural network and SVM to detect foreign substances. Moghadas divides the foreign substances into 4 groups and recognizes surface objects, foreign substances and bubbles efficiently without rotating the bottles. However, this method may miss objects which falls to the bottom before detection. Xiao *et al.* [7] detects movement by calculating the difference among three contiguous frames, tracks the movement by Particle Filter (PF) and recognizes foreign substances based on the direction of movement. This method does well in slowly-moving objects while it is weak in computation and fast-moving objects.

In this paper, we developed different methods corresponding to different foreign substances. Moving-object Clustering (MC) is proposed to detect subsiding-slowly objects. Clustering [8] is an unsupervised learning method, breaking up a set of observations into clusters in which observations are similar in some sense. In other words, clustering is to make observations in same cluster to be as similar as possible and the ones in different clusters to be as different as possible. Therefore we employ several features to calculate the similarity of moving object and cluster the similar ones of each frame into one cluster. After clustering, we develop some criterions to distinguish foreign substances from clusters.

Features of subsiding-fast objects may change rapidly, which reduces the precision of clustering. In this case, we calculate the frame difference between two frames to recognize foreign substances. We analyze the frame difference values of different samples to support our method.

The paper is organized as follows. Section 2 introduces the framework and architecture of the detection system. Section 3 presents the classification of foreign substances. Section 4 presents algorithm of subsiding-slowly foreign substances detection. Section 5 proposes algorithm of subsiding-fast foreign substances detection. Section 6 is devoted to the experiments and analyses of the results. Section 7 concludes this paper.

## 2. Framework and Architecture

**Figure 1.**
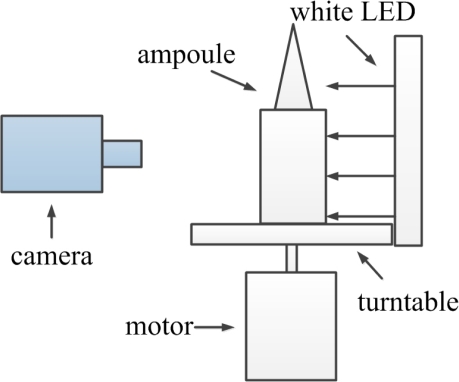
Framework of the detection system.

In order to gain relative movement and reduce the appearance of bubbles, we spin ampoule at high speed and then stop it suddenly. In this case, we can distinguish moving foreign substances from static ampoule bottles. An ampoule filled with injection is fixed on a turntable driven by a motor at speed 2,000 *r/min*. We use a white LED back-light illumination because white light is insensitive to the color of injection and back-light illumination can cause obvious contrast between transparent and opaque region. A sequence of frames are captured by an industrial camera with resolution of 2,048 × 1,536. The framework of the detection system is shown in Figure 1.

The overall process of the detection system framework is as follow:

1. High speed rotating. An injection ampoule is delivered to a turntable by conveyor belt and fixed on it. The turntable is driven by a motor located underneath and revolves rapidly together with the ampoule. A vortex is formed so that the foreign substances can move to the center of the ampoule. The ampoule rotates steady to reduce the probability of the appearance of bubbles.
2. Sudden stoping. After high-speed rotation for a few seconds, the movement is stopped suddenly. Due to inertia, foreign substances continue moving for a while and have relative movement from the ampoule bottle. Thus before the liquid slows down, the white LED is lit on and the camera begins to capture a sequence of frames as inputs of our detection algorithm.

## 3. Classification of Foreign Substances

**Figure 2.**
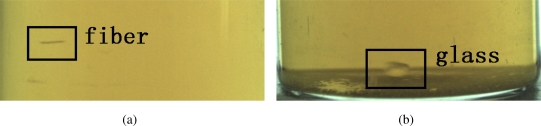
Foreign substances. **(a)** Fiber, **(b)** Glass.

Foreign substances used to be classified into black and white ones [3,4,7,9] when both back-light and bottom-light illumination are used together. In our framework, we only use back-light illumination and propose a new classification of foreign substances: subsiding-slowly ones and subsiding-fast ones. This classification is based on the subsiding speed of foreign substances. Subsiding-slowly foreign substances generally include rubber, hair and fiber, and subsiding-fast foreign substances mainly are glasses and metals. Illustrations of foreign substances, fiber and glass, are shown in Figure 2. Due to their different subsiding speed, the features of the two kinds of objects are different.

### 3.1. Subsiding-Slowly Foreign Substances

**Figure 3.**
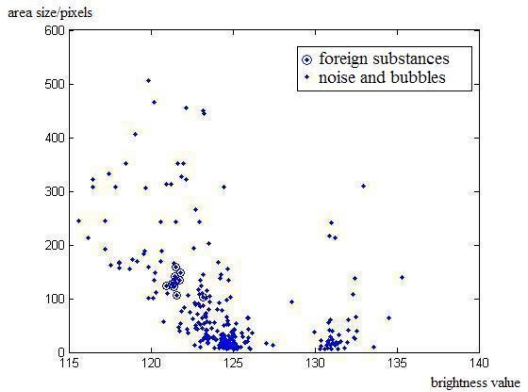
Distribution of brightness value and area size in a sequence of frames.

These foreign substances often have light weight and subside slowly after the ampoule stops moving. Therefore, we can assume that the average brightness value, area size and shape of the foreign substance are almost identical between contiguous frames. The distribution of brightness values and area sizes of the moving objects and the noise in a sequence of frames are shown in Figure 3, which is a statistical result after motion detection where moving objects and noise are obtained by considering the background image. The brightness values of those objects are within [115, 140], and area sizes are lower than 600. It is clear that brightness values and area sizes of the same foreign substance in a sequence of frames are close. As we assume the subsiding speed to be low, we use the positions of foreign substances between contiguous frames as another feature. Besides, Hu invariant moments [10] of foreign substance qualify as features for they are invariant to scale, rotation and reflection.

### 3.2. Subsiding-Fast Foreign Substances

In contrast to the subsiding-slowly objects, the features of subsiding-fast objects are that:

1. The density of subsiding-fast objects is relatively big, so the objects always move close to the bottom area of ampoule where bubbles hardly appear.
2. The shape of such object varies in frames.
3. They stop moving rapidly, generally in 3 or 4 frames.

**Figure 4.**
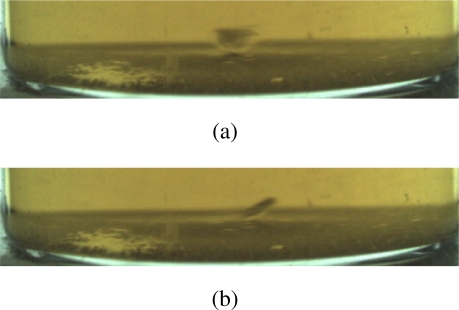
Two contiguous frames with glass. **(a)** Glass in frame *i*, **(b)** Glass in frame (*i* + 1).

Two contiguous frames with subsiding-fast foreign substance (glass) is shown in Figure 4. It is clear that the area size and shape of glass are different in two contiguous frames, hence clustering is not effective in detecting subsiding-fast foreign substances.

## 4. Detection of Subsiding-Slowly Foreign Substances

The detection of subsiding-slowly foreign substances includes three phases: motion detection, moving-object clustering and foreign substances recognition.

### 4.1. Motion Detection

Motion detection is to separate moving objects from frames. Images captured by camera include ampoule area and environment area, so we need to separate ampoule area from the image so as to reduce calculation. Since the height of ampoule and camera is fixed, we can set the vertical area into a fixed range. Compared with the vertical area, the horizontal area of ampoule varies in every detection. We employ Gaussian smooth and Canny operator to obtain the edges of ampoule, project the image to the horizontal direction, and gain the horizontal area by calculating the maximum value in the left half and right half part of the projection.

After separating the ampoule area from images, we obtain a static background by Algorithm 1. A pixel contributes to the static background if the difference between the brightness values of two contiguous frames is less than *δ*, then we add this pixel value to the sum *total*. Each pixel value of static background *BG*(*x, y*) is the mean of values added to *total*.

**Algorithm 1.** Static background gained from a sequence of frames.

**Input:**	
	*L* frames with *M* × *N* pixels;
	Brightness value of pixel (*x*, *y*) in frame *i*, *B_i_*(*x*, *y*);
**Output:**	
	Static background with *M* × *N* pixels, *BG*;
1:	**for** coordinate *x* = 0 to *M* − 1 **do**
2:	**for** coordinate *y* = 0 to *N* − 1 **do**
3:	*counter* = 0;
4:	*total* = 0;
5:	**for** frame *i* = 1 to *i* = *L* − 1 **do**
6:	**if** |*B_i_*(*x*, *y*) − *B*_*i*+1_(*x*, *y*)| < *δ***then**
7:	*counter* + +;
8:	*total* = *total* + *B_i_*(*x*, *y*);
9:	**end if**
10:	**end for**
11:	*BG*(*x*, *y*) = *total*/*counter*;
12:	**end for**
13:	**end for**
14:	**return***BG*;

As static background is obtained above, moving objects can be found by computing the difference between each frame and static background, as shown in Equation (1). Motion is described by a binary image *MT_i_* and a pixel is considered to be part of a moving object if its value equals to 1.

(1) $${\mathrm{MT}}_{i}(x,y)=\{\begin{array}{ll} 1 & \text{if}\left|F_{i}(x,y)-\mathrm{BG}(x,y)\right|\geq \delta \\ 0 & \text{others} \end{array}$$

At last, image morphological transformations is applied to reduce noise and fill up holds.

### 4.2. Moving-Object Clustering

Generally, based on whether a classification method classifies observations into a finite number of unrelated supervised classes or unsupervised clusters, the classification method is either supervised or unsupervised [11–14]. Clustering, also called exploratory data analysis, is unsupervised classification. The task of clustering is to split up a set of observations into clusters so that the similarity between observations within a cluster is larger than that between observations belonging to different clusters [11]. There are two types of clustering techniques: partitional clustering and hierarchical clustering [15,16].

**Figure 5.**
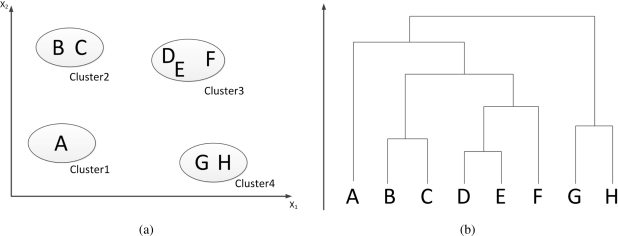
Illustration of hierarchical clustering algorithm. **(a)** Points fall into four clusters, **(b)** Dendrogram yielded by the algorithm.

Hierarchical clustering algorithms produce a nested series of partitions [8], find successive clusters using previously established clusters. Basically, these algorithms are either agglomerative (bottom-up) or divisive (top-down). Agglomerative algorithms separate each element as a cluster and then merge them into larger clusters. Divisive algorithms regard all the elements as a cluster and then divide it into smaller clusters. Figure 5 illustrates the operation of a bottom-up hierarchical clustering using a two-dimensional data set. Figure 5(a) describes 8 data (A–H) in four clusters. A dendrogram yielded by a hierarchical clustering algorithm represents the nested grouping based on the pattern and similarity of data, as shown in Figure 5(b). Different clusters can be obtained by breaking the dendrogram into different leaves [8].

Now come back to our detection, the proposed motion detection in Section 4.1 has marked all the suspected moving objects. After that, a hierarchical clustering algorithm is used to cluster the similarity of moving objects. Foreign substances recognition is then operated on the clusters.

As mention in Section 3.1, several features of subsiding-slowly foreign substances is almost invariant between contiguous frames. Moreover, our clustering algorithm is based on assumptions as follow: (1) The average brightness values, area sizes, Hu moments of one actual moving object in contiguous frames are analogous, and the positions of the object in contiguous frames are close. (2) The similarity of the features of the same actual object in the frames have negative correlation with the distance of the frames containing the object. (3) Suspected objects of one frame do not belong to the same actual object.

Based on the assumptions above and experiments, similarity between suspected objects *m* and *n* is defined in Equations (2)–(6):

(2) $$S_{B}=\frac{1}{1+\frac{\left|B_{m}-B_{n}\right|}{B_{m}}}$$

(3) $$S_{A}=\frac{1}{1+\text{log}|\frac{A_{m}}{A_{n}}|}$$

(4) $$S_{H}=\text{exp}(-\alpha \sum_{i=1}^{7}\left|H_{m}(i\right)-H_{n}(i)|)$$

(5) $$S_{P}=\text{exp}(-\beta \sqrt{{\left(x_{m}-x_{n}\right)}^{2}+{\left(y_{m}-y_{n}\right)}^{2}})$$

(6) $$S=\{\begin{array}{ll} \frac{\left(S_{B}\times S_{A}\times S_{H}\times S_{P}\right)}{\Delta F} & \Delta F>0\\ 0 & \Delta F=0 \end{array}$$

where *B* denotes the average brightness value, *A* is the area size, *H*(*i*) indicates the *i* moment of the 7 Hu ones, *α* and *β* are constant, *x* is the X coordinate of the position while *y* is the Y coordinate, and Δ*F* is the distance between two frames. *S_B_* denotes the similarity between average brightness values of object *m* and *n. S_A_* is the similarity of two area sizes. The similarity of Hu moments is denoted by *S_H_*. *S_P_* represents the similarity of two positions. These four similarities form the overall similarity *S* of two objects. The result *S* of Equation (6) is located in [0, 1], where the more similar two objects are, the bigger *S* is. Besides, we define *D_S_* as the distance function between two objects, as shown in Equation (7).

(7) $$D_{S}=1-S$$

The clustering algorithm is depicted as follow:

1. Suppose we have *L* contiguous frames, each frame *i*, *i* ∈ [1, *L*], has *N_i_* suspected objects, and each object *n_i_* in each frame has *K* features. Therefore, K-dimension eigenvector of object is represented by *O_in_* = {*f*_*i*,*n*,1_, *f*_*i*,*n*,2_, …, *f*_*i*,*n*,*K*_ }, where *f*_*i*,*n*,*K*_ is the feature *k* of object *n* in frame *i*. All the suspected objects form a matrix in Equation (8):
  (8) $$O_{S}=[O_{11},O_{12},\cdots ,O_{1N_{1}},O_{21},O_{22},\cdots ,O_{in_{i}},\cdots ,O_{LN_{L}}]$$
  where *O*_*in*_*i*__ is the eigenvector of object *n* in frame *i*, *n_i_* ∈ [1, *N_i_*]. Table 1 shows the eigenvector *O*_11_.
2. Using *O_S_* as input, we operate bottom-up hierarchical clustering algorithm.

**Figure 6.**
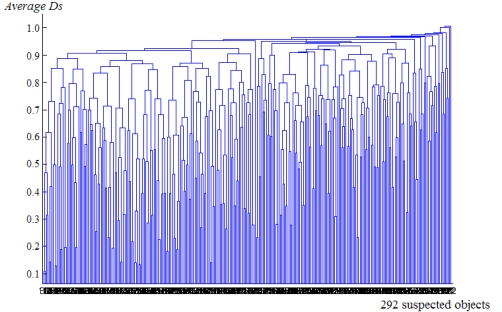
Dendrogram of one clustering on a sequence frames.

Figure 6 shows the dendrogram of one clustering on a sequence frames, the nested relationship between objects and the average *D_S_* in one cluster. There are 292 suspected objects. Although there are still 50 clusters where average *D_S_* is 0.85, only a few clusters have objects which appear in each frame. We can rule out most clusters based on this.

### 4.3. Foreign Substances Recognition

Now we have clusters about noise, bubbles, and foreign substances. Because foreign substances subside slowly, we can assume that these foreign substances and bubbles receive constant force and have uniformly accelerated motion, while the motion of noise is irregular. Furthermore, bubbles always float up to injection surface while foreign substances go down to bottom or travel smaller vertical distance than bubbles. Therefore, we can recognize foreign substances based on motion calculated as follow:

First of all, centroid of objects is gained by Equation (9):

(9) $$x_{c}=\frac{\sum_{(x,y)\in R}\mathrm{xB}(x,y)}{\sum_{(x,y)\in R}B(x,y)},\;\;\;\;\;y_{c}=\frac{\sum_{(x,y)\in R}\mathrm{yB}(x,y)}{\sum_{(x,y)\in R}B(x,y)}$$

where *B*(*x*, *y*) denotes the brightness value of pixel (*x*, *y*) in object area *R*. Assume the motion of an object has initial velocity (*v_x_*, *v_y_*), acceleration (*a_x_*, *a_y_*) and initial position (*x*_0_, *y*_0_), then the position of the object at time *t* is calculated by Equation (10):

(10) $$x(t)=x_{0}+v_{x}\times t+\frac{1}{2}a_{x}\times t^{2},\;\;\;\;\;\;\;\;\;y(t)=y_{0}+v_{y}\times t+\frac{1}{2}a_{y}\times t^{2}$$

It is obvious that the third-order derivatives of *x*(*t*) and *y*(*t*) are zero ideally. Though the actual third-order derivative is not zero due to inaccurate positions of objects, there is large difference between third-order derivative of actual objects and that of noise. Third-order derivative of discrete positions are shown in Equations (11) and (12).

(11) $$\frac{d^{3}x(t)}{{\mathrm{dt}}^{3}}=x(t+3)-3x(t+2)+3x(t+1)-x(t)$$

(12) $$\frac{d^{3}y(t)}{{\mathrm{dt}}^{3}}=y(t+3)-3y(t+2)+3y(t+1)-y(t)$$

In *L* frames, we can get (*L* − 3) third-order derivatives of object positions. Add the square of these derivatives up to be *T_D_* in Equation (13).

(13) $$T_{D}=\sum_{t=1}^{L-3}\left({\left(\frac{d^{3}x(t)}{{\mathrm{dt}}^{3}}\right)}^{2}+{\left(\frac{d^{3}y(t)}{{\mathrm{dt}}^{3}}\right)}^{2}\right)$$

A threshold *η* is set to recognize actual objects and noise. It is considered as an actual object if *T_D_* < *η* and as noise if otherwise. Besides, we use the distance on vertical direction to separate foreign substances and bubbles. A threshold *μ* is set to complete this job.

## 5. Detection of Subsiding-Fast Foreign Substances

As analyzed in Section 3.2, detection algorithm of subsiding-fast foreign substances is different from that of subsiding-slowly foreign substances. Due to the features of subsiding-fast objects, Frame Difference is proposed. Assume the bottom area of an ampoule in frame *i* is ***G****_i_* shown in Equation (14):

(14) $${\text{G}}_{i}=\left(\begin{array}{cccc} g_{00} & g_{01} & \cdots & g_{0(Q-1)}\\ g_{10} & g_{11} & \cdots & g_{1(Q-1)}\\ \vdots & \vdots & \ddots & \vdots \\ g_{(P-1)0} & g_{(P-1)1} & \cdots & g_{\left(P-1)(Q-1\right)} \end{array}\right)$$

in which *P* × *Q* denotes the bottom area size of an ampoule and *g_jk_* is the brightness value of pixel (*j*, *k*). We compute the sum of row vectors and that of column vectors respectively in *G_i_*, as shown in Equations (15) and (16):

(15) $$V_{i}=(\begin{array}{lllll} V_{i0} & V_{i1} & V_{i2} & \cdots & V_{i(Q-1)})=\sum_{j=0}^{P-1}\left(\begin{array}{lllll} g_{j0} & g_{j1} & g_{j2} & \cdots & g_{j(Q-0)} \end{array}\right) \end{array}$$

(16) $$W_{i}=(\begin{array}{lllll} W_{i0} & W_{i1} & W_{i2} & \cdots & W_{i(P-1)})=\sum_{k=0}^{Q-1}\left(\begin{array}{lllll} g_{0k} & g_{1k} & g_{2k} & \cdots & g_{(P-1)k} \end{array}\right) \end{array}$$

where ***V_i_*** is the sum of row vectors and ***W_i_*** is the sum of column vectors. Frame Difference *D* is defined in Equations (17)–(19). Unlike calculating the difference between pixels, we first derive the sum of row vectors ***V_i_*** and sum of column vectors ***W_i_***, then compute the differences of the sums between two contiguous frames, *D* is the sum of two differences.

(17) $$D_{i}=\left\|V_{i+1}-V_{i}\right\|+\left\|W_{i+1}-W_{i}\right\|$$

(18) $$\left\|V_{i+1}-V_{i}\right\|=\sum_{j=0}^{Q-1}{\left(V_{(i+1)j}-V_{\mathrm{ij}}\right)}^{2}$$

(19) $$\left\|W_{i+1}-W_{i}\right\|=\sum_{k=0}^{P-1}{\left(W_{(i+1)k}-W_{\mathrm{ik}}\right)}^{2}$$

**Figure 7.**
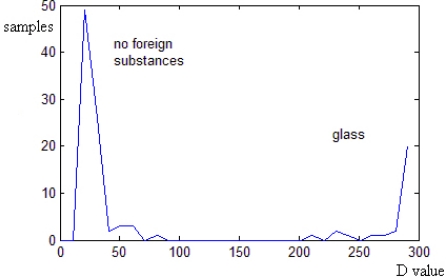
The statistical result of Frame Difference D.

After image preprocessing like Gaussian smoothing, we calculate Frame Difference *D*_1_ between frame 1 and frame 2, because the different between those frames may be the biggest among all contiguous two frames. At last, a threshold *θ* is set to recognize subsiding-fast foreign substances. In order to analyze the effect of *D*, we employ 112 ampoule samples among which 28 samples have glass. Figure 7 shows the statistical result of *D*. It is obvious that the *D* value of ampoules with subsiding-fast foreign substances is much bigger than that of ampoules without foreign substances. *θ* is set to be 150 to detect subsiding-fast foreign substances.

## 6. Experimental Result

**Figure 8.**
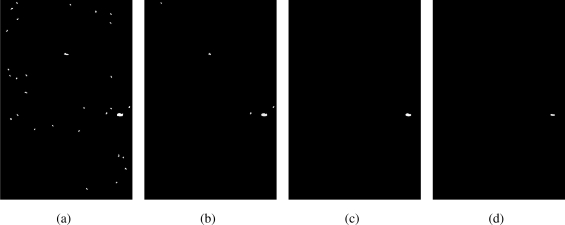
Experiment of foreground on different *δ*, foreign substance with the biggest white area. **(a)** *δ* = 1, **(b)** *δ* = 3, **(c)** *δ* = 5, **(d)** *δ* = 7.

Before testing our system, we have to solve a problem: decide the values of parameters mentioned above. *δ* in Equation (1) is used to separate foreground from background and decided by experiment. Figure 8 illustrates one of the experimental result. When *δ* = 1, there will be too much noise in foreground which only increases the computation of detection. When *δ* = 7, boundary area of foreign substance is lost which affects the features of foreign substances. We choose *δ* = 3 rather than *δ* = 5 to increase the precision of motion detection.

*α* and *β* are scale parameters used to reduce the contribution of Hu moments on similarity and adjust the pixel distance to the ampoule size in image. They are set to be $\frac{1}{30}$ and $\frac{1}{200}$.

Our method uses *μ* in Section 4.3 to recognize foreign substances and bubbles. According to Pan’s simulation [17], the speed of bubbles with over 40 *μm* radius rises to over 3 *mm/s* after 0.01 second. The camera captures 9 frames every second and 1 *mm* distance has 60 pixels. Assume that the speed of bubbles is 3 *mm/s*, so bubbles can move up at least 20 pixel between two frames. Since some foreign substances hardly move in frames, we set *μ* to be −10. If one object moves up less than 10 pixel, it is considered to be foreign substance.

**Figure 9.**
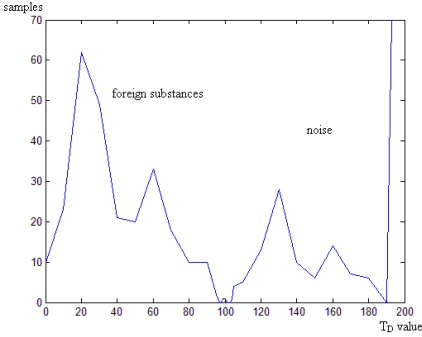
*T_D_* values of 603 track samples.

*η* in Section 4.3 is proposed to recognize foreign substances and noise. In order to decide the value of *η*, we compute *T_D_* values in Equation (13) using 603 trace samples as shown in Figure 9. 206 trace samples of foreign substances are used, whose *T_D_* values are all lower than 100. Over 99.7% percent of *T_D_* values of noise samples are higher than 100. It is obvious that *T_D_* can recognize traces of foreign substances and that of noise effectively, therefore we choose *η* to be 100.

After choosing the value of the parameters, some experiment are made and the results are shown in Tables 2 and 3. There are 200 ampoule samples, among which 129 samples are without foreign substance, 41 samples contain subsiding-slowly foreign substances and the others contain subsiding-fast foreign substances. The accuracy of recognizing subsiding-fast foreign substances is 96.67%. It is because some glass are big and heavy, and they stop moving before frames are captured. In this case, Frame Difference *D* is lower than *θ*. Some improvement about control system, motor and camera can solve this problem. Accuracy of subsiding-slowly foreign substances detection is 97.56%. The reasons for failed detection are that:

1. foreign substances move to the side wall of an ampoule and then miss in a frame;
2. noise due to camera and environment cause the failure;
3. foreign substances move too slowly, so they are considered to be background.

The detection precision of ampoules without foreign substance is 98.45%. Since the variation of brightness in some frames is apparent due to the camera and environment, ampoules without foreign substance is recognized into those with subsiding-fast foreign substances.

The comparison among our system and other systems is shown in Table 4. The qualified liquids’ detection accuracy of our system is the highest among the systems brought forward. The accuracy of our system in detecting unqualified liquids is the second best. It is shown that our system can detect qualified and unqualified injections effectively.

We apply Knapp–Kushner testing programs [18], which are accepted by U.S. Food & Drug Administration (FDA) and European pharmacopoeia, to test our automatic detection system. The testing samples are the same as those in Table 2. Because the judging criterion is *FQB/FQA >* 1, the result shown in Table 5 points out that our detection machine is more effective than workers.

## 7. Conclusions

This paper mainly studies the detection of foreign substances in injection ampoule. A mechanical system is designed and achieved. We build the system with an industry camera, a motor and some supporting tables like turntable. Foreign substances are classified into two categories: subsiding-slowly ones and subsiding-fast ones. Since we detect foreign substances based on movement, classifying those objects based on their movement features is reasonable. Different characteristics between those categories lead to different detection methods. Moving-object clustering is proved to be effective in detecting subsiding-slowly objects. Four features are employed to calculate the similarity between objects in clusters. Bubbles hardly affect the detection of subsiding-fast objects, so a light computation method Frame Difference is proposed.

Future work may focus on the improvement of mechanical system, algorithms and application of the system in production.

## Figures and Tables

**Table 1.** Eigenvector *O*_11_.

object	eigenvector										
	*B*	*A*	*x*	*y*	*H*[1]	*H*[2]	*H*[3]	*H*[4]	*H*[5]	*H*[6]	*H*[7]
*O*_11_	*f*_1,1,1_	*f*_1,1,2_	*f*_1,1,3_	*f*_1,1,4_	*f*_1,1,5_	*f*_1,1,6_	*f*_1,1,7_	*f*_1,1,8_	*f*_1,1,9_	*f*_1,1,10_	*f*_1,1,11_

**Table 2.** Detection result of 200 samples.

Category	Number of samples	True detection	Accuracy
Subsiding-fast	30	29	96.67%
Subsiding-slowly	41	40	97.56%
No foreign substance	129	127	98.45%

**Table 3.** Overview result of detection.

Total number of samples	200
Number of true detection	196
Detection accuracy	98.00%

**Table 4.** Comparison among our system and other systems.

System	Our system (200 samples)	Lu’ system [4] (180 samples)	Zhou’s system [5] (480 samples)	Xiao’s system [7] (3,500 samples)
Detection accuracy of qualified liquids	98.45%	96.10%	97.00%	98.32%
Detection accuracy of unqualified liquids	97.18%	91.80%	98.89%	96.00%

**Table 5.** Knapp–Kushner testing result.

FQA^1^	FQB^1^	FQB/FQA	Criterion	Result
407	613	1.506	*FQB/FQA >* 1	Detection machine is more effective than workers

1 *FQ_i_*: quality factor of bottle *i*. *FQ_i_* = (*n/N*) × 10, where n is unqualified times of bottle i, N is total testing times of bottle i. *FQA: FQ* of workers. *FQA* = *FQA*_[7,10]_ = Σ *FQA_i_*, only *FQA_i_* located in [7, 10] are added. *FQB: FQ* of detection machine. *FQB* = *FQB*_[7,10]_ = Σ *FQB_i_*, only *FQB_i_* located in [7, 10] are added.
